# Effect of Integrated Training on Balance and Ankle Reposition Sense in Ballet Dancers

**DOI:** 10.3390/ijerph182312751

**Published:** 2021-12-03

**Authors:** Chai-Wei Lin, Yu-Lin You, Yi-An Chen, Tzu-Chan Wu, Cheng-Feng Lin

**Affiliations:** 1 Department of Biomedical Engineering, College of Engineering, National Cheng Kung University, Tainan 701, Taiwan; noletalin@gmail.com (C.-W.L.); oilfish@bme.ncku.edu.tw (Y.-L.Y.); 2 Department of Physical Therapy, Shu-Zen Junior College of Medicine and Management, Kaohsiung 821, Taiwan; 3 Department of Physical Therapy, College of Medicine, National Cheng Kung University, Tainan 701, Taiwan; manmanjump@gmail.com (Y.-A.C.); tzuchanwu@gmail.com (T.-C.W.); 4 Physical Therapy Center, National Cheng Kung University Hospital, Tainan 701, Taiwan

**Keywords:** plyometric, proprioception, core stability, ankle sprain

## Abstract

Objective: To investigate the effects of a 6-week integrated training program on the ankle joint reposition sense and postural stability in ballet dancers. Methods: Sixteen female ballet dancers participated in the study and underwent a 6-week integrated training program consisting of plyometric, proprioception and core stability exercises along with a home program involving additional ankle muscle strengthening and stretching. The ankle joint reposition tests and the parameters of the center of pressure (COP) while performing *grand-plie* (deep squatting) and *releve en demi-pointe* (standing on balls of foot) movements were measured before and after training. Results: After 6 weeks, participants showed significantly smaller absolute ankle joint reposition errors in dorsiflexion (*p* = 0.031), plantarflexion (*p* = 0.003) and eversion (*p* = 0.019) compared to the pre-training measurement. Furthermore, after training, a significantly slower average COP speed at pre-equilibrium during *grand-plie* movement (*p* = 0.003) and pre-equilibrium phase of *releve en demi-pointe* (*p* = 0.023) were observed. In addition, the maximum COP displacement in the medial-lateral direction was significantly smaller at pre-equilibrium phase during *grand-plie* (*p* = 0.044) and *releve en demi-pointe* movements (*p* = 0.004) after training. Conclusions: The 6-week integrated training program improved the ankle joint reposition sense and postural control in the medial-lateral direction during *grand-plie* and *releve en demi-pointe* movements.

## 1. Introduction

Ballet requires intensive training to reach the demanded aesthetic standards. Consequently, ballet dancers are under intense physical and psychological demands during training, rehearsal and performance [1]. The physical demands imposed on dancers are exacerbated by insufficient warm-up or cool-down periods, inadequate stretching exercises, and deficient training on core muscles [2]. Such improper practices may lead to micro-trauma of the soft tissues (i.e., the ligaments, tendons, or muscles). Furthermore, the accumulation of these micro-traumas over time increases the risk of injury and may potentially affect the dancer’s performance, or even end their career [3]. Many studies have shown that ballet dancers are at a greater risk of injury to the ankle or foot than other performers or athletes due to the higher demands placed on the ankle and foot during the execution of ballet movements [2,4,5]. Among all the injuries suffered by ballet dancers, one of the most common injuries is ankle sprain [6], accompanied by residual symptoms such as pain, muscle weakness, delayed neuromuscular response, and impaired proprioception function. Thus, proper training programs aimed at minimizing ankle injuries and improving the balance ability are essential in maintaining the health and performance of ballet dancers.

Proprioception plays an essential role in ensuring a smooth and coordinated movement of the body by providing a conscious or unconscious awareness of the joint position [7]. Proprioception of the ankle joints is particularly important to ballet dancers in maintaining postural stability in response to perturbations or challenging movements (e.g., single-leg standing or jump-landing). Consequently, proprioception training is widely applied to ballet dancers with ankle sprain in order to improve or regain the function of the proprioception receptors around the ankle joint [7,8,9,10].

Plyometric exercise is an intensive exercise consisting of fast and powerful movements achieved via eccentric contraction of the muscles followed immediately by quick concentric contraction. The effects of plyometric exercise on performance and injury prevention have been widely studied in many sports, including soccer, basketball and volleyball [11,12,13]. Brown et al. [9] compared the effects of six-week plyometric training and six-week traditional weight training in basketball players and found that both training programs improved muscle strength of the lower extremities and power-related variables. However, the effects of plyometric exercise on balance control were not investigated. Additionally, athletes who received six-week plyometric training had significant improvement on the knee joint reposition sense compared to those receiving regular training [14,15]. The balance ability and the functional performance [15,16,17], such as single leg hopping distance [16] or standing jump distance [17] were also improved significantly after plyometric training in athletes. Plyometric exercise of the lower extremities generally involves repetitive jump-landing movements and agility tasks with rapid changes of directions [10,18]. Such movements involve instantaneous stretch-shortening cycles that actuate the mechanical or proprioception receptors on the ligaments of the foot or tendons around the ankle joint due to the muscle spindle is more sensitive to the instantaneous stretching [19], and are thus of great potential benefit in improving the reposition sense of the ankle joint and enhancing the balance ability accordingly.

Core stability exercise (or core strengthening) is a widely used training program for improving athletic performance or preventing injury [20]. A greater stability of the core region provides the foundation for an improved force generation and transition of the extremities, and therefore plays a key role in improving postural stability [21,22] and sports performance [23,24,25].

As described above, the ankle joint in ballet dancers is subjected to intense physical demands during the execution of ballet movements. Consequently, effective training programs are essential to prevent injuries to the ankle joint (particularly strains) and improve the balance ability accordingly. Previous studies have shown that plyometric exercise, proprioception training and core stability strengthening are effective techniques for minimizing the risk of injury and improving performance in many different types of sports [26,27,28]. The present study speculates that such training programs may also benefit ballet dancers. Accordingly, an integrated six-week ballet training program is devised consisting of plyometric exercise, proprioception training and core stability exercise. The effectiveness of the proposed program is evaluated by measuring the center of pressure (COP) parameters of ballet dancers while performing *grand-plie* (deep squatting) and *releve en demi-pointe* movements before and after the training program, respectively. It is hypothesized that the ballet dancers will exhibit an improved ankle joint reposition sense and improved balance ability during ballet movements after receiving the proposed training program.

## 2. Materials and Methods

### 2.1. Participants

Sixteen female ballet dancers having ballet training history of minimum seven years and routine ballet training of minimum two hours per week were recruited from a college dance department to participate in this study. The mean age was 17.50 ± 1.37 years. The mean body height and body weight was 161.44 ± 4.22 cm and 51.03 ± 4.52 kg. The body mass index was 19.59 ± 1.82 kg/m^2^. All of the dancers had a classical ballet training history of at least seven years (10.72 ± 2.92 years).

Participants with a history of balance and vestibular problems, or any other impairment which might potentially affect their balance, or any acute inflammation or pain that affected their performance at the physical screening stage, were excluded.

In addition, four participants reported previous ankle sprain more than 4.5 years ago and did not have another onset of ankle sprain since then. The study was designed in accordance with ethical protocols and all the participants read and signed a formal consent form approved by the university Institutional Review Board (ER-95-190) prior to joining the study.

### 2.2. Assessments

#### 2.2.1. Joint Reposition Sense

The joint reposition sense of the ballet dancers was evaluated using a bi-axis electrogoniometer (GA 150, Biometrics Ltd., Gwent, UK) placed around the ankle joint. The electrogoniometer signals were recorded by instruNet software through an analog-to-digital box (instruNet-iNet-100, Omega Enginerring Inc., Norwalk, CT, USA).

The absolute errors of active ankle joint reposition sense were measured in target angles of 10° dorsiflexion (DF), 20° plantarflexion (PF), 10° eversion (EV), 10° inversion (IV) and 20° inversion in the dominant leg. Note that the dominant leg was determined as the leg used preferentially by the participants in performing forward hops.

In conducting the measurement process, the dancers sat on a custom-made chair with hip and knee flexion at 90° and trunk erected. The axis of motion in the sagittal plane (i.e., dorsiflexion and plantarflexion) was taken as the line passing through the lateral and medial malleolus with the ankle in the neutral position. By contrast, the motion of the ankle in the frontal plane (i.e., eversion and inversion) was tested with the ankle in slight plantarflexion. The participants were asked to close their eyes during the tests to avoid visual cues. The participant’s ankle was placed passively to the target angle and maintained at the placed angle for 5 s. The ankle was then repositioned passively to the initial position by the same investigator. Once the ankle was restored to the initial position, the participant was instructed to reposition the ankle actively to the target angle and maintain the ankle in that position for 5 s.

#### 2.2.2. Balance Ability during Ballet Movements

The balance ability test was measured during ballet dancing movements including *grand- plie* and *releve en demi-pointe*. The three-dimensional trajectories of the reflective markers placed on the sacrum and heel posteriors of participants were captured at a sampling rate of 100 frames per second by a videographic acquisition system consisting of eight CCD cameras (Eagles, Motion Analysis Corporation, Rohnert Park, CA, USA) in order to define the phases during movement. In addition, the center of pressure (COP) positions of the participants’ feet were detected by a force plate (9281B Kistler Instrument Corp., Winterthur, Switzerland) at a rate of 1000 samples per second. The videographic data and force plate data were time-synchronized to 1000 Hz using a linear interpolation method. The participants were asked to stand on a large piece of paper in the ballet first position and the outlines of the feet were then drawn on the paper. The foot length was defined as the distance between the tip of the second toe and the mid-heel position. Similarly, the *turnout* angle was defined as the angle between the lines constructed by the second toes and mid-heels of the two feet [29].

**Figure 1:**
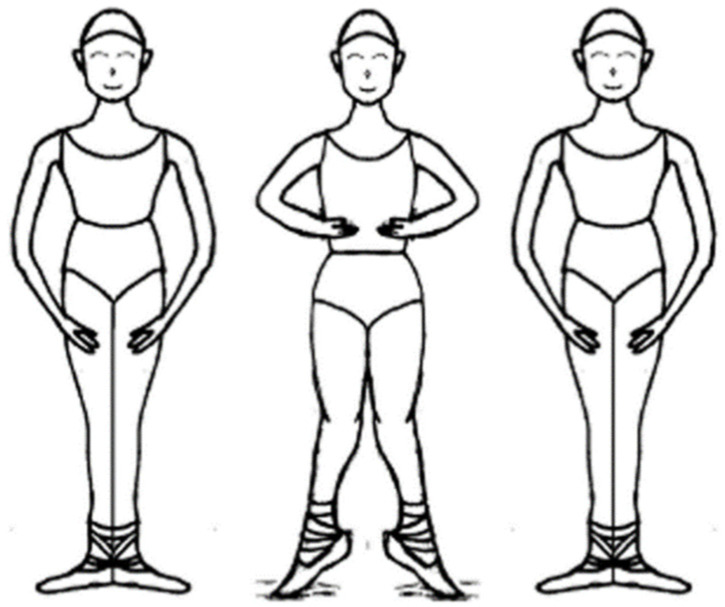
Releve en demi-pointe.

**Figure 2:**
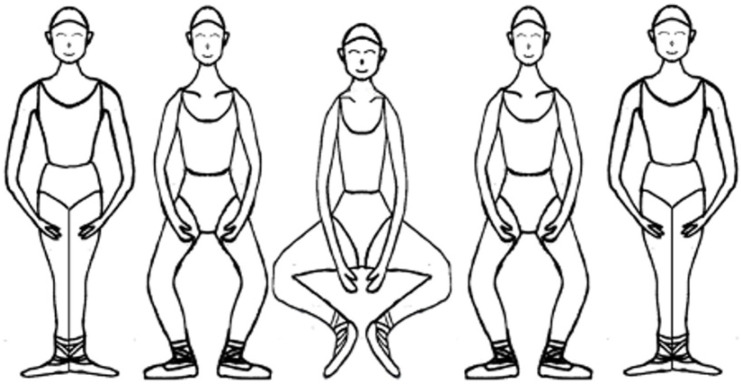
Grand-plie.

The participants put on ballet slippers and then performed 20-s *releve en demi-pointe* and 20-s *grand-plie* movements on the force plate. The ballet *releve en demi-pointe* movement commenced from the ballet first position and involved raising the heels up to *demi-pointe* within 5 s, maintaining this position for 10 s, and then returning to the initial position within 5 s (Figure 1). The *grand-plie* movement also began from the ballet first position, and involved deep squatting with heel-off within 5 s, maintaining the deep squatting position for 10 s, and then returning to the initial position within 5 s (Figure 2). For all movements, auditory cues were delivered by a metronome to control the movement speed. In addition, three successful trials were obtained for all movements.

### 2.3. Training Session

The six-week integrated training program consisted of plyometric exercise, proprioception training, and core stability training (1 h per day, 3 days per week) (Table 1). In every training session, the participants were asked to warm up with 5 min cycling at a self-selected speed followed by bilateral hamstring and calf muscle stretching. The participants had training sessions in the laboratory every week and received additional home training programs consisting of gastrocnemius and hamstring stretching, ankle muscle strengthening, core muscle training, and plyometric exercises (Table 2). Each dancer was given a daily log to document their compliance to the home training program and investigators reviewed the daily log every week to ensure their home training volume.

### 2.4. Procedure

Participants received joint reposition sense tests and balance ability tests during ballet movements. They then underwent a six-week training program and home training program. The measurements (joint reposition sense, balance ability test during ballet movements) were performed again after a 6-week training.

### 2.5. Data Reduction and Analysis

The COP-related parameters were used to represent the balance ability during *grand-plie* and *releve en demi-pointe* movements.

For analysis purposes, the *grand-plie* movement was divided into five phases, namely a 5 s lowering phase from the initiation of movement to the lowest sacrum position; a 10 s squatting phase (divided into three equal sub-phases: pre-equilibrium, equilibrium and post-equilibrium) with the sacrum markers in the lowest position; and a 5 s rising phase from the squatting position to the original standing position. The *releve en demi-pointe* movement was similarly divided into five phases, namely rising, pre-equilibrium, equilibrium, post-equilibrium, and lowering based on the position of the sacrum marker.

The COP positions were determined from the virtual acting points of the ground reaction forces. The average speed of the COP in each phase of the movement was computed as the total trajectory length traveled by the COP divided by the corresponding time. The ellipse area was determined as the area enclosing 95% of the COP positions, and was formed by linear curve fitting of the axes. The COP displacements were normalized to the corresponding *turnout* angle (*θ*) and average foot length (*l*) to eliminate the influence of inter-participant differences, i.e.,

$$NCOP_{AP}=\frac{COP_{AP}}{l\times \cos\frac{\theta }{2}},\;NCOP_{ML}=\frac{COP_{ML}}{l\times \sin\frac{\theta }{2}},$$

where *NCOP_AP_* denotes the normalized *COP* displacement in the anterior-posterior direction, while *NCOP_ML_* denotes the normalized *COP* displacement in the medial-lateral direction. The *COP* parameters for analysis included the average *COP* speed, the *COP* 95% ellipse area, the normalized maximal *COP* displacement, and the standard deviation (SD) of the *COP* displacement in the anterior-posterior and medial-lateral directions in each phase of the corresponding ballet movement. The SD of *COP* displacements throughout the ballet movement was calculated to assess the variability of *COP* displacement within a trial.

### 2.6. Statistical Analyses

The variables of interest included the absolute errors of the active joint reposition sense of the ankle and the aforementioned COP measures. The Shapiro–Wilk test was used to test the normality of the outcome measures. The paired-t test was then performed to test the training effects on the errors of joint reposition sense and balance ability during ballet dance performance. The effect sizes were also calculated to evaluate the strength of comparison. The significance level was set as 0.05 in every case. All of the statistical analyses were performed using commercial SPSS software (Vs. 17.0; SPSS Inc., Chicago, IL, USA).

## 3. Results

### 3.1. Joint Reposition Sense

After training, the absolute repositioning errors in 10° dorsiflexion (*p* = 0.031), 20° plantarflexion (*p* = 0.003) and 10° eversion (*p* = 0.019) were improved significantly. However, no significant difference was observed between the pre- and post-training repositioning errors in 10° and 20° inversion (Table 3).

### 3.2. Balance Ability during Ballet Movements

During *releve en demi-point*, participants showed a slower average COP speed in the rising (*p* = 0.011), pre-equilibrium (*p* = 0.023) and equilibrium phases after training. Participants also showed a smaller maximum displacement in the medial-lateral direction during the pre-equilibrium phase (*p* = 0.004) and a smaller SD of the COP displacement in the anterior-posterior direction during the lowering phase (*p* = 0.034). However, no significant difference was observed in the 95% COP ellipse area, maximum displacement in the anterior-posterior direction, or SD of the displacements in the medial-lateral direction after training (Table 4).

During *grand-plie*, participants showed a slower average COP speed in the lowering (*p* = 0.018), pre-equilibrium (*p* = 0.003), equilibrium (*p* = 0.009) and post-equilibrium (*p* = 0.011) phases after training. Participants also showed smaller maximum displacements of the COP in the medial-lateral direction during the lowering (*p* = 0.004), pre-equilibrium (*p* = 0.044) and post-equilibrium (*p* = 0.011) phases. A smaller SD of the displacements in the medial-lateral direction during the lowering (*p* = 0.002), pre-equilibrium (*p* = 0.014), equilibrium (*p* = 0.046) and post-equilibrium (*p* = 0.017) phases were observed after training. However, no significant difference was observed between the pre- and post-training values of the 95% COP ellipse area, maximum displacement of the COP, and SD of the COP displacements in the anterior-posterior direction (Table 5).

## 4. Discussion

### 4.1. Ankle Joint Reposition Sense

The improvement in the reposition sense can be attributed most likely to the elements in the 6-week integrated training program involving dorsiflexion movements (e.g., deep squatting) on the foam mat or air discs. The execution of dorsiflexion movements on such unstable surfaces actively triggers the proprioceptive mechanoreceptors around the ankle joint and gives rise to an improved joint reposition sense as a result. Similarly, the plyometric training exercises involved continuous jump-landing tasks, in which the dancers were required to react quickly in the landing period, thereby actuating the proprioception function of ankle dorsiflexion to enhance the pre-stretching effect of the muscles and increase the contraction force for the following jump motion. Thus, it appears that both plyometric training and proprioception exercises are beneficial in improving the ankle joint reposition sense in ankle dorsiflexion.

Routine ballet training in itself (i.e., no other additional training) appears to be sufficient to improve the repositioning ability of the ankle in dorsiflexion, plantar flexion and eversion. A previous study investigated the ankle joint reposition ability of gymnasts and non-gymnasts and found that the gymnasts had a superior reposition ability performance [18]. It was suggested that this was the natural consequence of the long-term effect of gymnast training [30]. Other studies have suggested that a superior joint replication ability may be associated particularly with movements such as single-leg standing; characterized by extreme ankle plantarflexion [19]. Ballet dancers’ ankle joints are often required to bear the entire weight of the body in extreme positions, such as *demi-pointe* (i.e., standing on the metatarsal heads) or full pointe (i.e., standing on tip-toes). Standing in these extreme positions requires full ankle plantarflexion combined with slight ankle eversion to maintain stability. Consequently, the medial side of the forefoot takes a larger proportion of the weight bearing. This in turn suppresses lateral deviation of the COP and reduces the risk of lateral ankle sprain. Overall, therefore, the improved reposition sense in ankle dorsiflexion, plantarflexion and eversion observed in both groups in the present study may be the natural result of regular ballet training rather than the integrated training program.

No improvement was observed in the ankle joint reposition sense in inversion following the training process. Notably, this contradicts the hypothesis of the present study that the training program can prevent episodes of ankle sprain. It is speculated that the insignificant training effect may be the natural result of regular ballet training. In particular, when performing heel-rise movements in ballet pointe shoes, ballet dancers are instructed to balance on the tips of two or three toes with the foot positioned away from the ankle inversion position. The dancers may apply the similar strategy during training sessions. Consequently, the ankles are routinely placed either neutrally or at slight eversion, and hence the effect of training on the ankle inversion reposition sense is inevitably reduced.

Plyometric exercise involves two main adaptations, namely peripheral and central. In peripheral adaptation, the stretch sensitivity of the Golgi tendon organs (GTOs) is heightened, and hence the muscle spindle contributes more afferents to the central nervous system, together with better proprioception [26]. The continuous jumping movements prescribed in the current training program involve the gastrocnemius muscle as the primary muscle. In other words, the muscle spindles of the gastrocnemius are the primary stimulated targets. This may explain the improved postural stability of the dancers observed in the medial-lateral direction after 6-week training since the plantarflexor and dorsiflexor with *turnout* (with both hips externally rotated with the heels in contact with each other) are the primary muscles responsible for controlling postural stability in medial-lateral sway during *grand-plie* and *releve en demi-pointe* movements.

Central adaptation results from the anticipatory movements required to execute the plyometric exercises prescribed in the training program. For example, the preparation for serial jumping movements facilitates the activity of the central nervous system and hence enhances the sensitiveness of the muscle spindles to position changes. The facilitation of muscle spindles may improve the balance ability during ballet movements and joint reposition ability as a previous study showed that the ankle plantar flexor torques is associated with postural stability [31]. The improved ankle reposition in dorsiflexion and plantarflexion again may contribute to dancers’ postural stability in ballet movements. Furthermore, relatively challenging ballet movements with *turnout* compared to quiet standing are a way to challenge proprioceptive awareness, as a previous study showed that quiet standing is less challenging for dancers [32].

### 4.2. Balance Ability during Ballet Movements

The effects of training on the postural stability of the ballet dancers were observed by measuring the speed and displacements of the COP during *releve en demi-pointe* and *grand-plie* movements. The dancers showed a slower COP average speed after the training program; thereby indicating a more stable posture. The improved postural stability may stem from a greater core stability and improved joint reposition sense. For example, a previous study investigated the transient effect of core stability exercises on postural sway, and found that core stability exercises reduce the range of COP displacements in the medial-lateral direction and decrease the COP speed during quiet standing [22]. Furthermore, the co-contraction of the back extensor muscles and abdominal muscles induced in the core exercises in the training program may result in an improved torso stability, which also tends to reduce the COP speed during the ballet movements [33]. In addition to a slower COP speed, the ballet dancers also showed an improved joint reposition sense after training. In a previous study, it was shown that participants with functional ankle instability (FAI) exhibited a poorer ankle joint reposition sense and greater COP displacement [34], and thus, the poor ankle joint reposition sense is related to greater COP displacement. The improved joint reposition ability after training in our study inferred that dancers may have superior proprioceptive awareness to detect the changes of postural stability during ballet movements.

The dancers had both a lower absolute displacement of the COP in the medial-lateral direction and a smaller SD of the COP displacements after the training program during *releve en demi-pointe* and *grand-plie* movements. Previous studies have reported that improved postural stability in the medial-lateral direction but not in the anterior-posterior direction is associated with the use of different balance strategies in the two directions. In particular, in side-by-side stance, balance in the anterior-posterior direction is maintained mainly by the ankle joint, whereas in the medial-lateral direction, balance is maintained predominantly by the hip abductor/adductor [35]. The core stability exercises prescribed in the integrated training program increase the muscle strength around the hip joints. Consequently, both the absolute values of the COP displacement in the medial-lateral direction and the SD values improve significantly after training.

A previous meta-analysis review reported that proprioception exercises can improve subjective instability and functional outcomes such as Star Excursion Balance test [36]. Similar improvement was also found in our study: ballet dancers in the current study showed improved balance ability when performing ballet movements after integrated training. The neuromuscular control for movements that require precision and postural control ability required the proprioception to contribute to the motor programing [37]. The joint reposition sense was positively correlated with the ability of postural control [34]. This represented that individuals with better joint reposition sense may demonstrate better postural control ability. Hence, the improved ankle joint reposition sense after training may contribute to the improvement of the balance ability during ballet movements. On the other hand, the smaller maximum COP displacement observed in the medial-lateral direction after training may be correlated with the improved reposition sense of dorsiflexion and plantarflexion caused by the more sensitive detection of ankle motion with the foot in the *turnout* position [38].

### 4.3. Limitations

In the present study, participants underwent assessments at pre- and post-training, however, this study did not have a control group to compare with the intervention effects. Hence, the intervention effectiveness is limited due to the training session including both the proposed training program and the regular ballet training.

## 5. Conclusions

This study has investigated the effectiveness of an integrated training program involving proprioception, plyometric and core stability exercises on the joint reposition senses of the ankle joint, the balance ability during specific ballet movements.

The integrated training program improved the neuromuscular control of the ankle joint, which may minimize the risk of ankle sprain during ballet movement by improving the joint reposition sense in dorsiflexion, plantarflexion, and eversion and the balance ability during specific ballet dance movements. Thus, the proposed integrated training program may be an option to be included in ballet dancers’ training program on improving the neuromuscular control of the ankle joint and the balance ability.

## Figures and Tables

**Table 1:** Exercise program.

Week	Proprioception Training	Plyometric Exercise	Core Stability	Others
1	Standing on the foam with demi-plie & *grand-plie* (10)	Line jump (forward-to-backward, and side-to-side) (20 ∗ 2)	Abdominal bracing (10 s ∗ 10) Bridging exercise with one foot on the air bag and one foot on the mat (10 ∗ 2) Swiss-ball (basic bounce, heel raising bounce, and toe raising bounce) (30)	Towel squeezing (3 s ∗ 20)
2	One-leg-standing on the foam with Arabesque (10 ∗ 2). Two-leg-standing on wobble board (clockwise and counter-clockwise) (5)	Two-legged jump from mat to 10-cm stage with firm surface (forward-to-backward, and side-to-side) (20)	Bridging exercise with each foot on air bag (10 ∗ 2) Swiss-ball exercise (alphabetic sitting on mat, bouncing with front and side foot tap) (30)	Towel squeezing (4 s ∗ 20)
3	One-leg-standing on foam with free-leg with sand bag and alphabetic movement (1 ∗ 2) Two-leg-standing on wobble board (clockwise and counter-clockwise) (5)	Two-leg jump from mat to 15-cm stage with firm surface (forward-to-backward, and side-to-side) (20) Ankle jump (40)	Bridging exercise with crossed-leg on the air bag (10 ∗ 2) Swiss-ball exercise (leg march on ball with each bounce, and alphabetic standing on foam)	Towel squeezing exercise with 1 kg sandbag (5 s ∗ 20)
4	One-leg-standing on mat with eyes-closed Standing on the pair of air-bags in 2nd position and perform the demi-plié	Two-leg jumping from mat to 15-cm stage with mat surface (forward-to-backward, and side-to-side) (20) Ankle jump (20) Scissors jump (10)	Bridging exercise with figure-four-leg on the air bag Swiss-ball exercise (alphabetic sitting on airbag, hop around ball)	Towel squeezing exercise with 1.5 kg sandbag (6 s ∗ 20)
5, 6	One-leg-standing with eyes-closed on foam (10 s ∗ 6) Standing on the two air-bags and *grand-plie* (10) Catch sandbag while standing on the BOSU (week 5: 1 kg sandbag; week 6: 3 kg sandbag) (30)	One-leg jumping from firm surface to 10-cm stage (10) One-leg continuous jump in S-shape (10) Squat-tuck jump (10) Cone jump with 3 cones (forward and side-to-side) (5 ∗ 2)	Bridge exercise with limb movement with BOSU beneath back (10 ∗ 2) Elbow-support on BOSU with trunk straight (week 5: double-leg support, week 6: one-leg support) Swiss-ball (alphabetic sitting with one foot on BOSU (2), full jumping jacks (30), sitting skier (30))	Towel squeezing exercise with 2 kg (week 5: 7 s ∗ 20; week 6: 8 s ∗ 20)

BOSU: Both Sides Up balance trainer.

**Table 2:** Home program.

Week	Flexibility	Muscle Strength of Ankle	Deep Muscle of Foot	Core Stability	Balance/Agility
1	Gastrocnemius stretch (30 s ∗ 9) Hamstring stretch (30 s ∗ 9)	Ankle invertor, evertor, dorsiflexor, plantarflexor with orange thera-band (5 s ∗ 30)	Towel squeezing (3 s ∗ 30)	Abdominal hollowing (10 s ∗ 30)	NA
2	Same as week 1	Same as week 1	Towel squeezing with 0.5 kg weight on towel (3 s ∗ 30)	Abdominal hollowing (10 s ∗ 30) Cat-camel exercise (60)	NA
3	Same as week 1	Ankle invertor, evertor, dorsiflexor, plantarflexor with green theraband (5 s ∗ 30)	Same as week 2	Cat-camel exercise (60)	NA
4	Same as week 1	Same as week 3	Same as week 2	Bird-dog exercise (10 s ∗ 30)	Deep squatting with *turnout* (15 s ∗ 15) Single-leg standing with arm motion (15 s ∗ 15)
5	Same as week 1	Ankle invertor, evertor, dorsiflexor, plantarflexor with blue theraband (5 s ∗ 30)	Same as week 2	Supine with reciprocal leg motion (60) Side bridge (45 s ∗ 30)	NA
6	Same as week 1	Same as week 5	Same as week 2	Supine with reciprocal leg motion (60) Side bridge (45 s ∗ 30)	Lateral jumping (30)

**Table 3:** Mean ± SD of absolute error of active joint reposition sense in dominant-leg (°).

		Training Group	*p*-Value	Effect Size
10° dorsiflexion	PRE	3.13 ± 3.77	0.031	0.311
	POST	1.69 ± 1.26		
20° plantarflexion	PRE	4.57 ± 3.18	0.003	0.422
	POST	2.72 ± 1.98		
10° inversion	PRE	1.57 ± 1.16	0.232	0.173
	POST	1.38 ± 1.10		
20° inversion	PRE	1.78 ± 1.34	0.162	0.202
	POST	1.94 ± 1.31		
10° eversion	PRE	1.43 ± 1.35	0.019	0.339
	POST	0.91 ± 0.69		

PRE: pre-training; POST: post-training; 0.5 ≤ Effect size < 0.8: medium effect; Effect size ≥ 0.8: large effect.

**Table 4:** COP parameters during *releve en demi-pointe* (mean ± SD).

Phase		Average Speed (cm/s)	95% Ellipse Area (cm^2^)	Normalized Maximum Displacement		SD of Displacement	
				Anterior-Posterior	Medial-Lateral	Anterior-Posterior	Medial-Lateral
rising	PRE	**9.650** ± **2.335**	1.700 ± 0.731	0.200 ± 0.088	0.083 ± 0.032	0.083 ± 0.032	0.034 ± 0.010
	POST	**8.474** ± **0.895**	1.626 ± 0.622	0.181 ± 0.067	0.070 ± 0.023	0.077 ± 0.032	0.030 ± 0.009
	Effect size	0.24	0.47	0.19	0.47	0.19	0.42
pre-equilibrium	PRE	**9.440** ± **3.041**	1.303 ± 1.071	0.132 ± 0.043	**0.078** ± **0.042**	0.063 ± 0.025	0.030 ± 0.015
	POST	**8.220** ± **1.434**	0.917 ± 0.461	0.119 ± 0.045	**0.063** ± **0.023**	0.054 ± 0.022	0.023 ± 0.008
	Effect size	0.30	0.44	0.38	0.44	0.38	0.58
equilibrium	PRE	8.550 ± 2.737	0.726 ± 0.468	0.117 ± 0.060	0.061± 0.034	0.050 ± 0.018	0.022 ± 0.009
	POST	7.580 ± 1.090	0.719 ± 0.441	0.114 ± 0.055	0.051± 0.016	0.051 ± 0.027	0.019 ± 0.006
	Effect size	0.05	0.38	0.04	0.38	0.04	0.39
post-equilibrium	PRE	8.231 ± 2.666	0.638 ± 0.472	0.109 ± 0.033	0.058 ± 0.029	0.048 ± 0.017	0.021 ± 0.008
	POST	7.564 ± 1.193	0.702 ± 0.388	0.107 ± 0.037	0.052 ± 0.020	0.048 ± 0.018	0.020 ± 0.008
	Effect size	0.06	0.24	<0.01	0.24	<0.01	0.13
lowering	PRE	10.460 ± 2.616	2.543 ± 1.499	0.229 ± 0.126	0.115 ± 0.048	**0.094** ± **0.049**	0.043 ± 0.015
	POST	10.268 ± 2.803	2.453 ± 1.719	0.213 ± 0.102	0.114 ± 0.062	**0.076** ± **0.029**	0.042 ± 0.019
	Effect size	0.14	0.02	0.45	0.02	0.45	0.06

COP: center of pressure; SD: standard deviation; PRE: pre-training; POST: post-training; values in bold of difference between test-time indicate significant differences (*p* < 0.05); 0.5 ≤ Effect size < 0.8: medium effect; Effect size ≥ 0.8: large effect.

**Table 5:** COP parameters during *grand-plie* (mean ± SD).

Phase		Average Speed (cm/s)	95% Ellipse Area (cm^2^)	Normalized Maximum Displacement		SD of Displacement	
				Anterior-Posterior	Medial-Lateral	Anterior-Posterior	Medial-Lateral
lowering	PRE	**10.770** ± **2.467**	4.037 ± 2.255	0.329 ± 0.100	**0.126** ± **0.049**	0.144 ± 0.044	**0.050** ± **0.019**
	POST	**9.507** ± **1.608**	3.368 ± 1.454	0.300 ± 0.083	**0.100** ± **0.030**	0.132 ± 0.045	**0.038** ± **0.009**
	Effect size	0.61	0.35	0.32	0.64	0.27	**0.81**
pre-equilibrium	PRE	**8.474** ± **2.575**	0.847 ± 0.447	0.122 ± 0.047	**0.061** ± **0.022**	0.054 ± 0.023	**0.026** ± **0.009**
	POST	**7.028** ± **0.765**	0.684 ± 0.344	0.113 ± 0.039	**0.052** ± **0.014**	0.045 ± 0.016	**0.021** ± **0.006**
	Effect size	0.76	0.41	0.21	0.49	0.45	0.65
equilibrium	PRE	**8.073** ± **2.621**	0.528 ± 0.315	0.106 ± 0.036	0.051 ± 0.022	0.043 ± 0.015	**0.021** ± **0.009**
	POST	**6.757** ± **0.754**	0.523 ± 0.331	0.099 ± 0.031	0.045 ± 0.014	0.028 ± 0.017	**0.017** ± **0.006**
	Effect size	0.68	0.02	0.21	0.33	0.94	0.52
post-equilibrium	PRE	**7.950** ± **2.658**	0.512 ± 0.316	0.095 ± 0.034	**0.053** ± **0.020**	0.040 ± 0.016	**0.021** ± **0.008**
	POST	**6.726** ± **0.732**	0.501 ± 0.271	0.096 ± 0.033	0.043 ± 0.013	0.043 ± 0.015)	**0.017** ± **0.008**
	Effect size	0.63	0.04	0.03	0.59	0.19	0.50
rising	PRE	10.079 ± 2.604	2.674 ± 1.596	0.246 ± 0.095	0.099 ± 0.042	0.116 ± 0.049	0.043 ± 0.018
	POST	9.745 ± 3.989	3.900 ± 5.164	0.280 ± 0.108	0.111 ± 0.142	0.133 ± 0.056	0.042 ± 0.031
	Effect size	0.10	0.32	0.33	0.11	0.32	0.04

COP: center of pressure; SD: standard deviation; PRE: pre-training; POST: post-training; values in bold of difference between test-time indicate significant differences (*p* < 0.05); 0.5 ≤ Effect size < 0.8: medium effect; Effect size ≥ 0.8: large effect.

## Data Availability

Data from this study are available from the corresponding author on reasonable request.
